# Ten-year predictors of major adverse cardiovascular events in patients without angina

**DOI:** 10.4102/safp.v65i1.5629

**Published:** 2023-08-04

**Authors:** Preesha Premsagar, Colleen Aldous, Tonya Esterhuizen

**Affiliations:** 1 Department of Internal Medicine, Nelson R. Mandela School of Medicine, University of KwaZulu-Natal, Durban, South Africa; 2 Department of Clinical Medicine, Nelson R. Mandela School of Medicine, University of KwaZulu-Natal, Durban, South Africa; 3 Division of Epidemiology/Biostatistics, Department of Global Health, Faculty of Medical and Health Sciences, Stellenbosch University, Stellenbosch, South Africa

**Keywords:** Cox model, diabetes, Indian, morbidity, mortality

## Abstract

**Background:**

Longstanding cardiovascular risk factors cause major adverse cardiovascular events (MACE). Major adverse cardiovascular events prediction may improve outcomes. The aim was to evaluate the ten-year predictors of MACE in patients without angina.

**Methods:**

Patients referred to Inkosi Albert Luthuli Hospital, Durban, South Africa, without typical angina from 2002 to 2008 were collected and followed up for MACE from 2009 to 2019. Survival time was calculated in months. Independent variables were tested with Cox proportional hazard models to predict MACE morbidity and MACE mortality.

**Results:**

There were 525 patients; 401 (76.0%) were Indian, 167 (31.8%) had diabetes at baseline. At 10-year follow up 157/525 (29.9%) experienced MACE morbidity, of whom, 82/525 (15.6%) had MACE mortality. There were 368/525 (70.1%) patients censored, of whom 195/525 (37.1%) were lost to follow up. For MACE morbidity, mean and longest observation times were 102.2 and 201 months, respectively. Predictors for MACE morbidity were age (hazard ratio [HR] = 1.025), diabetes (HR = 1.436), Duke Risk category (HR = 1.562) and Ischaemic burden category (HR = 1.531). For MACE mortality, mean and longest observation times were 107.9 and 204 months, respectively. Predictors for MACE mortality were age (HR = 1.044), Duke Risk category (HR = 1.983), echocardiography risk category (HR = 2.537) and Ischaemic burden category (HR = 1.780).

**Conclusion:**

Among patients without typical angina, early ischaemia on noninvasive tests indicated microvascular disease and hyperglycaemia, predicting long-term MACE morbidity and MACE mortality.

**Contribution:**

Diabetes was a predictor for MACE morbidity but not for MACE mortality; patients lost to follow-up were possibly diabetic patients with MACE mortality at district hospitals. Early screening for ischaemia and hyperglycaemia control may improve outcomes.

## Introduction

In a December 2020 statement, the World Health Organization declared that coronary artery disease (CAD) had remained the leading cause of death globally for the last 20 years. Furthermore, CAD had accelerated by more than 2 million cases since 2000 to nearly 9 million cases in 2019. Coronary artery disease now represents 16% of total deaths from all causes.^1^

Modifiable and nonmodifiable risk factors precede the onset of CAD. The role of these cardiovascular risk factors has emerged as a central mechanism in developing cardiovascular disease. It has been established that atherosclerosis is a disease of multiple aetiologies, caused by an intricate cascade of molecular and cellular changes in the intima of the arterial wall. Modifiable risk factors may include diabetes, hypertension, dyslipidaemia, smoking, increased body mass index (BMI) and increased waist circumference (WC). Nonmodifiable risk factors may include age, gender, race and family history. Once a critical level of luminal stenosis in the artery has occurred, the result is a demand–supply mismatch in oxygenated blood flow, resulting in angina.^2,3,4^

Typical angina, also known as chronic stable angina (CSA), is a signal of the early presence of CAD, which progressively worsens with effort as the lumen becomes more stenotic.^2^ The three cardinal criteria to define typical angina were described in 1768 by William Herbenden, an English physician, and are still used today. These three criteria are:

- precipitated by exertion or emotion
- sited in the anterior chest, neck, shoulders, jaw and left arm
- prompt relief by rest, relaxation or glyceryl trinitrate spray or trinitrotoluene.^3,4,5,6,7^

Patients with cardiovascular disease risk factors or typical angina have a high possibility of developing major adverse cardiovascular events (MACE). Major adverse cardiovascular events are a consequence of accumulative risk factors over time, resulting in long-term morbidity and mortality. Most studies define MACE as sustained angina pectoris, acute coronary syndrome (ACS), coronary revascularisation, cerebrovascular accident (CVA) and congestive cardiac failure (CCF). Major adverse cardiovascular events cause substantial overall disease burden measured in disability-adjusted life-years lost. This also incurs high costs in medical care for patients, their families and society. Not all patients survive the consequences of MACE, and death as a composite was measured in landmark studies.^3,8,9,10^

Major adverse cardiovascular event prediction is therefore an important and widely studied subject. It has a significant impact on interventional decision-making, both for care and treatment of patients at risk and resource allocation. Extensive research to predict future events has been undertaken. Traditionally, many MACE prediction tools were based on scoring systems designed to forecast the event from known associate risk factors (predictors).^3^ Such predictive tools include the Global Registry of Acute Coronary Events (GRACE) and the Thrombolysis in Myocardial Infarction (TIMI) score. The GRACE is a simple, accurate and widely used risk-prediction tool validated in multiple populations. The GRACE uses clinical and laboratory features to forecast MACE and was included in European and American clinical guidelines on ACS management.^11^ The TIMI score is a simple prognostic scoring system to determine the patient’s risk for death or ischaemic event. The TIMI score uses age over 65 years, cardiac enzymes and coronary angiography features to predict MACE and was designed to allow for intervention.^12^

Studies also apply regression models and time-to-event analysis for secondary prevention.^13^ The Cox regression model has demonstrated that the risk factors independently associated with MACE are triple vessel disease, stent implantation, hypertension, renal impairment and increased uric acid.^8^ Cox regression also demonstrated that ageing and early insignificant coronary stenosis were strongly associated with future long-term MACE.^9^ In a local South African study that assessed the long-term outcome, Indian people had multiple risk factors for ACS, which contributed to the increased incidence of CAD in younger patients (< 45 years old). Family history of vascular disease had a strong association with the presence of ACS.^14^

Typical angina carries a higher likelihood of CAD (likelihood ratio 5.6). Atypical or noncardiac chest pain carries a lower likelihood of CAD (likelihood ratio 1.3).^3,4^ Therefore, there is a paucity of studies on MACE in patients who initially presented without typical angina. This study’s scientific value was to help understand the long-term MACE morbidity and MACE mortality in South African patients without typical angina at the onset. This knowledge allows earlier diagnosis, management with better outcomes and lower costs to patients and the healthcare system, adding social value. While other studies have examined tools that are widely used,^11,12^ this study aimed to evaluate the predictive factors of MACE specifically in South African patients without typical angina in a 10-year time-to-event follow-up. Granular information on the local population regarding important predictive factors was identified early on to allow for better control or intervention. Since CVD accounted for 16% of total deaths from all causes in 2020,^1^ steps may be taken to prevent the morbidity, mortality and economic burden associated with MACE.

## Method

### Study design

This observational study at Inkosi Albert Luthuli Central Hospital (IALCH), Durban, South Africa was retrospective from January 2002 to December 2008 and prospective from January 2009 to April 2019. Inkosi Albert Luthuli Central Hospital is a tertiary and quaternary hospital using electronic healthcare records to capture, store, access and manage patient records. Permission to access the databases was granted by the hospital (site) manager.

### Data collection

Patients with suspected CAD (chest pain and risk factors) or confirmed CAD (ACS, enzyme leak, ST deviation on electrocardiograph [ECG]) were referred from the base hospitals in the catchment areas (mainly Addington Hospital, R.K. Khan Hospital, Mahatma Gandhi Memorial Hospital and Prince Mshiyeni Hospital, but also other parts of KwaZulu-Natal and Eastern Cape) to the Department of Cardiology at IALCH. Depending on the presentation at the base hospital, patients were referred for outpatient evaluation for suspected CAD and inpatient evaluation for confirmed CAD, because angiography and intervention were immediately indicated.

Speedminer (Speedminer Sdn Bhd, Petaling Jaya, Malaysia) was the data warehouse and business performance management software package used by IALCH to manage, process and categorise the data collected on its patient databases. With Speedminer, all patients who attended IALCH Cardiology in the study period were able to be identified by their KwaZulu (KZ) numbers. Files of the identified patients were accessed on the two electronic databases used at IALCH. These were Medicom Clinician Access (CA) Live and the Sorian package developed by Siemens.

**FIGURE 1:**
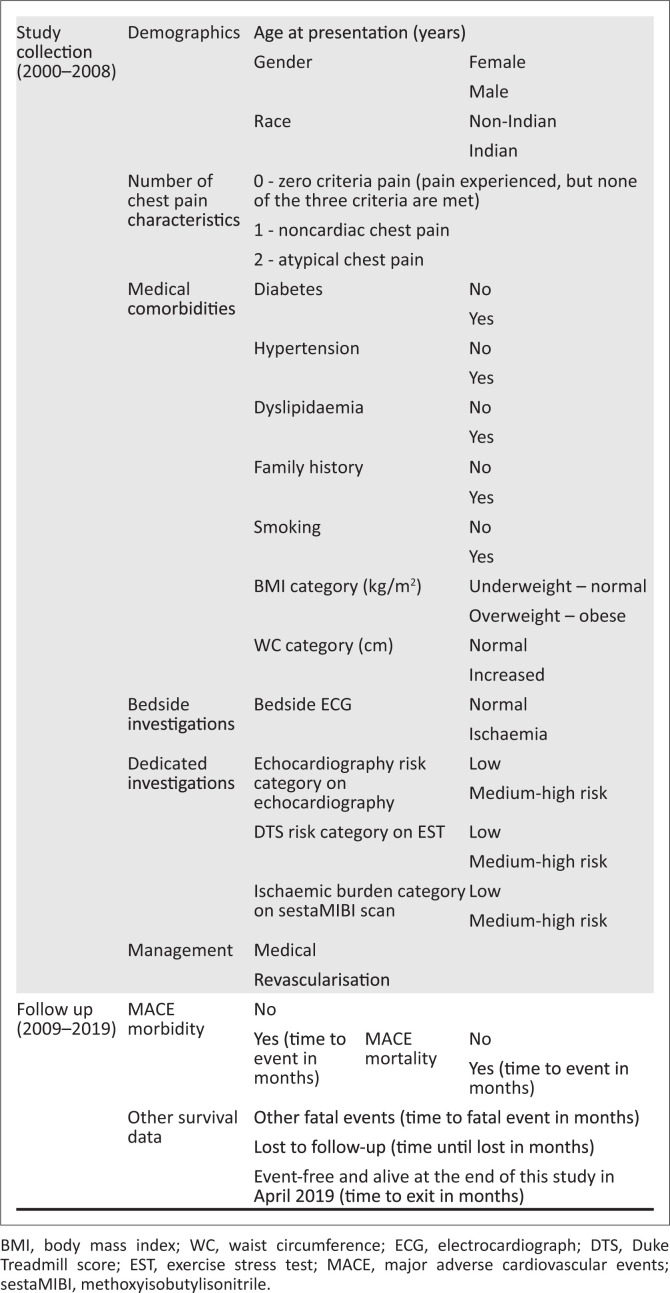
Data collected included.

Every patient referred to the IALCH Department of Cardiology from January 2002 to December 2008 was assessed for their symptoms, and those without typical angina were collected for the cohort; these were outpatients initially. Since typical angina was not present, patients were classed according to the symptoms they did have. If two out of three criteria were present, they had atypical chest pain; patients with one out of three criteria had noncardiac chest pain.^3,6,7^ Patients whose chest pain matched none of angina criteria^5^ were classed as zero-criteria chest pain. The data collected included demographics, medical comorbidities, bedside ECG^3,15^ and results of dedicated investigations performed in the 2002–2008 timeframe (Figure 1).

The dedicated investigations were accessed where available and scored using quantifiable, standardised scoring systems for objective evaluation. The first was the exercise stress test (EST). The Duke Treadmill Score (DTS) was used to objectively measure the exercise time, dynamic ECG changes and symptoms on the EST. The DTS was calculated, then categorised for DTS risk categories into low risk (above 5), medium risk (+4, –10) and high risk (–10).^16^ Echocardiography was also performed. It provided information on the left ventricular wall function through visual assessment for normal function or features suggestive of contractile dysfunction (hypokinesis, akinesis, dyskinesis and aneurysmal segment). The global average echocardiography score (GAES) was calculated, then categorised for echocardiography risk category into normal/low risk (below 1), small infarct/medium risk (1–2) and complications/high risk (above 2) (Figure 1).^17^

SestaMIBI (methoxyisobutylisonitrile) myocardial perfusion used 15 mCi of technetium 99m sestamibi to assess myocardial perfusion with a single-photon emission computerised tomography gamma camera. The summed difference score (SDS) was calculated, then categorised for ischaemic burden into normal/low risk (1–3), medium ischaemia (4–11) and severe ischaemia (above 13).^18,19^ Management of patients was recorded. Mostly, this was conservative; however, some underwent revascularisation before 2008. These data were captured accordingly and follow-up continued until 2019 for further MACE (Figure 1).

Patients were required to list three contact numbers on the filing system upon registry first at IALCH, and update contact details at follow-up visits where necessary. If the first contact number was unsuccessful, the second and third were accessed. However, in some cases, listed contact numbers led to family members, friends, neighbours or ex-employers who were able to provide additional contact numbers, and these numbers were pursued. Patients were deemed lost to follow-up if no positive contact was made after three (or more) attempts on all three numbers (and additional ones) over a 3-month period. Follow-up data collected as MACE included angiography for symptoms, diagnosis (ACS, CCF, arrhythmia, peripheral vascular disease [PVD] and CVA and intervention, revascularisation, pacemaker, peripheral vascular surgery) (Figure 1).

### Survival time

The age of each patient at the time of presentation between January 2002 and December 2008 was calculated in years. The patients were followed up for MACE morbidity and MACE mortality in the 10 years from January 2009 until April 2019.

For MACE morbidity, the survival time (months), as the response variable, was considered from the first presentation to the occurrence of *the first* MACE (fatal or nonfatal). Censoring was applied to the event-free patients at the end of the follow-up period in April 2019 who were lost to follow-up before the end without an event occurring or died from an unrelated event.

For MACE mortality, the survival time (months), as the response variable, was considered from the first presentation to the occurrence of mortality from MACE. Censoring was applied to the patients who did not have a MACE mortality at the end of the follow-up period, were lost to follow-up before the end or died from an unrelated event.

### Statistical analysis

The data were analysed with Stata version 16.1 for Windows (StataCorp LLC, College Station, Texas, United States). An alpha value of 0.05 was used to indicate statistical significance. The Cox proportional hazard model was used to identify the baseline factors from 2002 to 2008 associated with the occurrence of MACE morbidity and MACE mortality during follow-up from 2009 to 2019. Each independent variable was tested individually with the crude Cox model, and those variables with a *p*-value ≤ 0.2 were advanced into the adjusted model. The adjusted estimates of the independent variables were tested using manual intelligent modelling to determine the most parsimonious model. Variables with a *p*-value > 0.05 were dropped from the model one at a time, starting with the least statistically significant variable. The model was re-evaluated at each step, and a final model was declared once all remaining independent variables were statistically significant (*p* < 0.05). The proportional hazards assumption was tested for goodness of fit with Schoenfeld’s global test for assumption. The adjusted hazards ratios and 95% confidence intervals were reported in the final model for MACE morbidity and MACE mortality. Health records were electronically stored; missing data were accounted using the imputation method.

### Ethical considerations

This study was approved by the Biomedical Research Ethics Committee of the University of KwaZulu-Natal Nelson R. Mandela School of Medicine (ref. no. BE513/17).

## Results

From 2002 to 2008, there were 10 362 patients referred to IALCH for a variety of cardiac problems, including suspected CAD, but also valvular heart lesions, cardiomyopathy, pericardial disease, myocardial disease, conductive defects and many others. Of these 10 362, there were specifically 5378 patients referred for suspected or confirmed CAD. From the 5378, there were 564 without typical angina, suspected of having CAD and evaluated initially as outpatients. Of these, 39 were found to have established CAD and underwent previous bypass surgery (in private settings or at Wentworth Hospital) and were excluded from the study. The remainder was a cohort of 525 patients collected from 2002 to 2008.

In the cohort, there were 401 (76.0%) Indian patients and 167 (31.8%) diabetic patients at baseline on referral to IALCH. There were 26.0% (137/525) patients who were both Indian and diabetic at baseline. There were 157/525 (29.9%) patients who experienced MACE morbidity in the follow-up interval. Of these 157 patients, 82/157 (52.26%) patients experienced MACE mortality, either at the time of the *first MACE* or at a *consequential MACE* during the follow-up period (MACE mortality made up 15.6% (82/525) of the cohort). The 82 MACE mortality patients included 49 ACSs, 15 CVAs, 19 CCFs and two terminal cardiac arrhythmias.

Censoring was applied to the remaining 368/525 (70.1%) patients. These included 153/525 (29.2%) patients who were followed up event-free to the end of this study until April 2019; 195/525 (37.1%) patients who were lost to follow-up before the end; and 20 (3.8%) patients who died from other causes. Of the 195 patients who were lost to follow-up, 69.2% (135/195) were Indian, 34.3% (67/195) were diabetic at baseline and 26.1% (51/195) were Indian and diabetic at baseline. Furthermore, of those lost to follow-up, 76.9% (150/195) had at least one screening test in a medium-high risk category at baseline. From the remaining 23.1% (45/195) patients who had all low-risk categories at baseline, only four were diabetic and Indian at baseline. The 20 patients with other terminal events included seven gastrointestinal tumours, seven lung tumours, one breast cancer, one Hodgkin’s lymphoma, one motor neurone disease complications and three trauma-related (unnatural) deaths.

### Evaluation of major adverse cardiovascular event morbidity

The total analysis time at risk under observation for MACE morbidity was 53 643 months. The mean time was 102.2 months, and the longest observed time was at 201 months. The crude Cox model was performed to test each independent variable individually against the outcome of time to MACE morbidity (*n* = 525) (Table 1).

**TABLE 1:** Crude Cox model for major adverse cardiovascular events-morbidity (*N* = 525).

Predictor	Hazard ratio	*z*	*P* > |*z*|	95% CI	Advance to adjusted model
Presentation age	1.026628	3.07	0.002	1.009529−1.044017	Yes
Gender	0.8779004	−0.80	0.423	0.6383825−1.207284	No
Race	1.343472	1.33	0.184	0.8687086−2.077703	Yes
Diabetes	1.503321	2.49	0.013	1.090834−2.071786	Yes
Hypertension	1.074447	0.43	0.671	0.771644−1.496073	No
Dyslipidaemia	1.333586	1.78	0.076	0.9706518−1.832225	Yes
Family history	1.181263	1.04	0.297	0.8635439−1.615878	No
Smoking	1.061818	0.36	0.722	0.7635007−1.476696	No
BMI category	1.194613	1.10	0.272	0.8700294−1.640291	No
WC category	0.9396125	−0.38	0.702	0.6831294−1.292393	No
Pain	1.226582	1.91	0.056	0.9947616−1.512425	Yes
Bedside electrocardiography	1.287251	1.58	0.115	0.9403545−1.762118	Yes
Duke risk category	1.702845	2.92	0.004	1.191041−2.434577	Yes
Echocardiography risk category	1.941446	1.82	0.069	0.9496842−3.968911	Yes
Ischaemic burden category	1.503742	2.46	0.014	1.087031−2.080197	Yes
Revascularisation	0.807923	−0.76	0.447	0.4659883−1.400764	No

BMI, body mass index; WC, waist circumference; CI, confidence interval.

After crude Cox analysis, presentation age, race, diabetes, dyslipidaemia, pain, bedside ECG, Duke risk category, echocardiography risk category and ischaemic burden category were advanced into an adjusted Cox model with *p*-values < 0.2.

After manual intelligent modelling was performed, the final Cox model was generated by selecting variables with *p*-value < 0.05. The Cox regression model showed that presentation age, diabetes, Duke risk category and ischaemic burden category were hazards for a MACE morbidity. The global test *p*-value was *p* = 0.9074; therefore, the null hypothesis that the hazards were proportional for this model was not rejected.

For every 1-year increase in presentation age, the hazard of developing MACE morbidity increased by 2.5%. People with diabetes had a 1.4-times higher hazard than patients without diabetes for developing MACE. Patients whose screening tests for CAD were performed in the decade prior showed that medium-high risk Duke category on EST had a 1.5-times higher hazard than the low risk for developing MACE morbidity. The medium-high risk ischaemic burden category on sestaMIBI scan also had a 1.5-times higher hazard than the low risk for developing MACE morbidity (Table 2).

**TABLE 2:** Final Cox proportional hazard for major adverse cardiovascular event morbidity (*N* = 525).

Predictor	Hazard ratio	*z*	*P* > |*z*|	95% CI
Presentation age	1.025219	2.65	0.008	1.006489−1.044297
Diabetes	1.436774	1.96	0.050	0.9998235−2.064684
Duke risk category	1.56262	2.35	0.019	1.076399−2.268473
Ischaemic burden category	1.531443	2.31	0.021	1.066879−2.198299

Note: Schoenfeld’s test of proportional-hazards assumption – Chi^2^ = 1.02; degree of freedom = 4; probability > chi^2^ = 0.9074.

CI, confidence interval.

### Evaluation of major adverse cardiovascular event mortality

The total analysis time at risk under observation for MACE mortality was 56 658 months. The mean time was 107.9 months, and the longest observed time was at 204 months. The crude Cox model was performed to test each independent variable separately with the outcome of time to MACE mortality (*N* = 525) (Table 3).

**TABLE 3:** Crude Cox model with independent variables for major adverse cardiovascular event mortality (*N* = 525).

Predictor	Hazard ratio	*z*	*P* > |*z*|	95% CI	Advance to adjusted model
Presentation age	1.04876	3.87	< 0.001	1.023786−1.074343	Yes
Gender	0.8901103	−0.52	0.606	0.5720901−1.384915	No
Race	1.055645	0.19	0.850	0.6024084−1.849885	No
Diabetes	1.847701	2.76	0.006	1.194752−2.857497	Yes
Hypertension	1.138618	0.55	0.581	0.7181446−1.805278	No
Dyslipidaemia	1.4942	1.77	0.077	0.9578827−2.330925	No
Family history	0.9478436	−0.24	0.810	0.613173−1.465177	No
Smoking	0.9869415	−0.06	0.956	0.6187854−1.574138	No
BMI category	1.15354	0.63	0.526	0.7416436−1.794197	No
WC category	0.938556	−0.28	0.778	0.6044589−1.457316	No
Pain	1.115526	0.75	0.456	0.8369591−1.486808	No
Bedside ECG	1.154285	0.65	0.519	0.7466653−1.784433	No
Duke risk category	1.859179	2.41	0.016	1.121627−3.081724	Yes
Echocardiography risk category	2.932418	2.51	0.012	1.2654960−6.795025	Yes
Ischaemic burden category	1.817331	2.61	0.009	1.160793−2.845205	Yes
Revascularisation	0.9328079	−0.19	0.852	0.4490665−1.937643	No

BMI, body mass index; WC, waist circumference; ECG, electrocardiograph; CI, confidence interval.

After crude Cox analysis, presentation age, diabetes, Duke risk category, echocardiography risk category and ischaemic burden category were advanced into the first adjusted Cox model.

The final Cox regression model showed that presentation age, Duke risk category, echocardiography risk category and ischaemic burden category were hazards for MACE mortality occurring. The global test *p*-value was 0.8547; therefore, the null hypothesis that the hazards were proportional for this model was not rejected.

For every 1-year increase in age, the hazard of developing MACE mortality increased by 4.4%. Patients whose screening tests for CAD were performed in the decade prior were robust predictors of death from MACE. The medium-high Duke risk category on EST test had a 1.9-times higher hazard than the low risk for developing MACE mortality. The medium-high risk in the echocardiography risk category was 2.5-times higher hazard than the low risk for developing MACE mortality. The medium-high risk ischaemic burden category on sestaMIBI scan also had a 1.7-times higher hazard than the low risk for developing MACE mortality (Table 4).

**TABLE 4:** Final Cox proportional hazard for major adverse cardiovascular event mortality (*N* = 525).

Predictor	Hazard ratio	*z*	*P* > |*z*|	95% CI
Presentation age	1.044395	2.99	0.003	1.015075−1.074562
Duke risk category	1.983997	2.28	0.023	1.101280−3.574246
Echocardiography risk category	2.537154	1.96	0.050	0.998694−6.445567
Ischaemic burden category	1.780439	2.06	0.039	1.028659−3.081648

Note: Schoenfeld’s test of proportional-hazards assumption – Chi^2^ = 1.34; degree of freedom = 4; probability > chi^2^ = 0.8547.

CI, confidence interval.

## Discussion

This study aimed to evaluate the predictive factors associated with MACE in South African patients without typical angina in a 10-year time-to-event follow-up. At baseline, the cohort of 525 patients, collected from January 2002 to December 2008, consisted of patients suspected of having CAD, based on demographics and risk factors, and referred for further evaluation. These patients were initially afforded a lower index of suspicion for CAD because atypical or noncardiac chest pain carries a lower likelihood of CAD than typical angina.^3,4^

It has been confirmed that people with diabetes may often present without typical angina when experiencing a MACE, including silent myocardial ischaemia or angina-equivalent dyspnoea. This may lead to a diagnosis being missed.^20^ In the follow-up for MACE from January 2009 to April 2019, the degree of morbidity and mortality and factors that may have been early predictors for MACE mortality and MACE morbidity were assessed.

The modifiable and nonmodifiable risk factors were applied to the model. The patients underwent noninvasive screening tests to evaluate their symptoms. Screening tests were scored and categorised, then built into the model. The final MACE morbidity model showed that age, diabetes, medium-high Duke risk category and medium-high ischaemic burden category were predictors. The final model for MACE morbidity showed presentation age, medium-high risk on Duke risk category, echocardiography risk category and ischaemic burden category were predictors.

### Predictors for major adverse cardiovascular events

For every 1-year increase in age, the hazard of developing MACE morbidity and MACE mortality increased by 2.5% and 4.4%, respectively. The proportional assumption for the model was not violated; therefore, the hazards for MACE morbidity and MACE mortality indicated that increasing age increases the risk.

Although race was not in the final model, the cohort consisted of a predominantly Indian race group. The majority of Indian South Africans today are descendants of indentured labourers, of both Dravidian and Aryan lineage, brought to Natal between 1860 and 1911 to develop the country’s sugar industry. Millions of Indian people around the world are able to trace their ancestry to the subcontinent. However, despite great cultural and geographical diversity, it has been long established that migrant Indian people have a high mortality from CAD compared to other local ethnic groups.^21,22^

Historical research shows that cardiovascular disease was higher in Indian populations that have emigrated from the Indian subcontinent.^21,22^ Early studies in 1990 demonstrated that Indian and white racial groups in South Africa had extremely high mortality rates from CAD. Approximately 50% of deaths from all causes were from diseases of the circulatory system in Indian patients over the age of 45 years. Diabetes mellitus was strongly associated with CAD.^22^

A further breakthrough study in 2002 investigated ACS in South African Indian people of different age groups. The study of 2290 patients admitted between 1996 and 2002 divided them into three age groups: young (< 46 years; 20%), middle-aged (> 45 to < 66 years; 59%) and old age (> 65 years; 21%). Triple-vessel disease was confirmed in 48% of those who underwent angiography, with 14% requiring CABG (coronary artery bypass graft). Young Indian patients in this study differed markedly from other young population groups with CAD because they frequently had premature atherosclerosis with diffuse and aggressive disease.^21^

The problem remains ongoing, as demonstrated in the Phoenix Lifestyle Project. It has been found that the magnitude of change in risk factor prevalence over the past two decades has been of epidemic proportions in South African Indian descendants compared to other Indian descendants with similar environmental exposure.^23^

### Revascularisation

A study on the 10-year clinical outcomes of intermediate lesions on angiogram showed better outcomes than severe lesions that were stented, suggesting the benefit of optimal medical therapy in a subset of patients.^24^ Revascularisation was not a predictor in the final models of the 10-year outcome of MACE morbidity and MACE mortality. The Bypass and Angioplasty Revascularisation Investigation (BARI) Trial was a large multicentre trial that recruited patients from 1988 to 1991 and then followed them up for MACE. The BARI Trial used myocardial infarction and cardiac mortality to evaluate the efficacy of revascularisation with CABG and percutaneous intervention (PCI) in 1829 patients post. The 5-year mortality of diabetic patients on drug therapy with multivessel disease was significantly greater in the PCI group than the CABG group.^10^ The 10-year follow-up found no significant long-term advantage in mortality or myocardial infarction associated with the initial management with PCI or CABG, except for diabetic patients where CABG increased survival.^25^ In the 10-year outcome, revascularisation was not a predictor, as aligned with the BARI Trial. However, the significance of prediction of MACE with diabetes was also a similar finding between this study and the BARI Trial.

### Diabetes

The differences observed between the diabetic and nondiabetic patients in the BARI study gave rise to further studies in the Bypass and Angioplasty Revascularisation Investigation Type 2 Diabetes (BARI 2D) trial. The BARI 2D Study, a multicentre landmark randomised trial, concluded that early revascularisation appears to reduce nonfatal myocardial infarctions, but not all causes of mortality, for patients who underwent CABG.^10,25^ This study found diabetes to be a predictor of MACE morbidity but not MACE mortality. The prior finding in this study was aligned with the BARI study but not the latter. However, it has been established in this study that the 37.1% (195/525) patients who were lost largely represent Indian people who were diabetic at baseline or had at least one screening test with medium-high risk, indicating microvascular disease. Therefore, the 37.1% (195/525) patients lost to follow-up over the 10 years may reasonably represent Indian people with diabetes who had MACE mortality at the district hospital level and were thereafter lost to this study.

### Scoring system categories derived from noninvasive tests

The DTS risk category from the EST, echocardiography risk category from the echocardiogram and the ischaemic burden category from the sestaMIBI scan were all useful predictors for MACE morbidity and MACE mortality.

Literature states that DTS was categorised to predict CAD and the 4 years’ predicted survival rate. A score in the low-risk category (above 5) was associated with a 4-year survival rate of 79%. A score in the medium-risk category (+4 to –10) predicted a 4-year survival rate of 85%. A score in the high-risk category (below −10) indicated a survival rate of 79% only.^16^ In this study, DTS was an accurate prediction even at 10 years. The medium-high risk group had a 1.5-times higher hazard for MACE morbidity and 1.9-times higher hazard for MACE mortality compared to the low-risk group. The EST is therefore an important test for long-term MACE assessment.

In this study, echocardiography did not predict MACE morbidity in the 10-year follow-up; however, the medium-high risk category had a 2.5-times higher hazard than the low-risk category for developing MACE mortality. Literature shows that reduced ejection fraction (calculated by end-systolic and end-diastolic dimensions) below 40% was established as an independent 5-year predictor of the combined endpoint of death from CCF.^26^ Research on patients with Type 1 diabetes shows that premature diastolic dysfunction on echocardiography suggested diabetic cardiomyopathy and was associated with a fourfold increase in CCF and atherosclerosis.^27^ Regional wall motion abnormalities have a 1.7-times increased risk for embolic CVA. Left ventricle thrombi overlying the ventricle wall segments with abnormal motion, were capable of embolism and resultant ischaemic CVA.^28^ The mechanisms of MACE mortality for ACS, CCF and CVA may be related to early findings on echocardiography, therefore medium-high risk GAES was a reliable predictor.

Research has repeatedly shown ischaemia to be a critical predictor of outcomes, associated with a 12-times increased risk of future MACE morbidity and MACE mortality compared to those patients without ischaemia.^27^ In this study, the medium-high risk ischaemic burden category on the sestaMIBI scan had a 1.7-times and 1.5-times higher hazard than the low risk for developing MACE mortality and MACE morbidity. People with diabetes had been proven to have substantially altered microvascular function. Microvascular dysfunction, identified as an ischaemic burden on sestaMIBI, worsens in people with diabetes, contributing to poorer outcomes.^29^ In a multicentre observational study, each level of ischaemia, as assessed quantitatively, was associated with a greater adverse prognosis amongst patients with diabetes relative to nondiabetic patients.^30^ In this study, the medium-high ischaemic levels were compared to low-risk or normal scans. Although diabetes may not have featured in the final MACE mortality model, the ischaemic burden as a predictor may indicate the hazard associated with poor glycaemic control in the cohort.

### Limitations and suggested further research

A limitation to this study was the reliance on the base hospital referring patients to IALCH for further assessment. Patients who were never referred would therefore not be part of the study.

The beginning of the survival time was taken at the first point of contact at IALCH and not at the onset of medical comorbidities, such as diabetes. Often, the diagnosis of medical comorbidity may be delayed, and in some cases, patients were diagnosed at IALCH.

Patients were evaluated at baseline for existing medical comorbidities and investigations. The follow-up was performed thereafter to evaluate for MACE morbidity and MACE mortality. The follow-up did not account for new diagnoses of risk factors (e.g. a newly diagnosed diabetic). The reason for this is to use the knowledge about the patient at baseline to learn about future outcomes. This, however, also has the disadvantage of not incorporating newer information about the patient’s evolving risk profile.

Another limitation was the inability to contact 195 patients who were lost to follow-up over the 10 years. Every attempt was made to trace all patients; however, despite best efforts, a large number were never found.

Current research focuses mostly on migrant Indian people compared to other local racial groups. Further research may be recommended for comparison between migrant Indian people and Indian people who still live on the Indian subcontinent.

It is also recommended that further studies be conducted to compare South African Indian people and other South African racial groups in a robust observational study (such as a cohort study) to determine if there is an increase in CAD or MACE amongst South African Indian patients with otherwise similar risk factors.

## Conclusion

This cohort selected patients who presented without typical angina but had modifiable and nonmodifiable risk factors for CAD. People with diabetes may often present without typical angina because of neuropathy.^20^ This study had a predominantly Indian cohort, a racial group in whom ground-breaking findings in previous studies confirmed the frequency of young, premature atherosclerosis with diffuse and aggressive disease.^21^

This study also observed that the presence of ischaemia detected on sestaMIBI was a strong predictor for MACE morbidity and MACE mortality; extensive research confirms that ischaemia was indicative of underlying microvascular disease and hyperglycaemia.^29,30^ Revascularisation did not influence MACE in any way in the decade-long follow-up. Landmark studies have confirmed that people with diabetes had better 5-year mortality on medical therapy than with revascularisation^10^ At 10 years, there was no benefit for survival from revascularisation, except for diabetic patients with CABG.^25^ Diabetes was a predictor of MACE morbidity but did not feature in the MACE mortality model. There was also a large portion of patients lost to follow-up, who therefore may reasonably represent people with diabetes who had MACE mortality at the district hospital level and were thereafter lost to this study.

This study concludes that diabetes and early baseline ischaemia on noninvasive tests are critical long-term predictors of MACE morbidity and MACE mortality. Patients with diabetes do not present with typical angina, and intervention may not alter long-term outcomes.^3,4,6,10,25^ The recommendation is first to implement early screening for ischaemia, wherever and whenever it may be accessible and affordable in the base hospital setting. The second recommendation is aggressive control of risk factors, especially hyperglycaemia, to improve the evolution of this disease process.
